# Supplementary material for: Partial verification bias correction using scaled inverse probability resampling for binary diagnostic tests
Source: PLoS One. 2025 Sep 26;20(9):e0321440. doi: 10.1371/journal.pone.0321440 (PMC12469146; doi:10.1371/journal.pone.0321440)
Supplement: Attachment — Submitted filename: Response to reviewers.docx [file pone.0321440.s001.docx]

**Response to reviewers**

PONE-D-25-12005

Partial Verification Bias Correction Using Scaled Inverse Probability Resampling for Binary Diagnostic Tests

Dear reviewers,

Thank you for the constructive comments given to the manuscript. We provide all responses to your comments in the table below:

| **Reviewer #** | **Comment** | **Response** |
| --- | --- | --- |
| 1 | 1-The summary should be expanded a bit. | This has been expanded to 300 words |
| 1 | 2- The last sentence in the summary is not understandable, it should be rewritten | This sentence has been rewritten as “Although the methods are computationally demanding, ...” |
| 1 | 3-The spelling of the “universiti” in the address section should be corrected.  9-The spelling of the word “universiti” in line 339 should be corrected. | This is the official name and spelling of the university (in Malay), which is indexed in academic databases. This should not be changed. |
| 1 | 4-The sentence starting with "in the study" on line 34 and the rest should be written in a new paragraph. | “In the study” refers to Arifin and Yusof’s study (preceding sentence). Therefore, the paragraph starting with “In the study” has been moved to a new paragraph and “In the study” has been rephrased as “In their study...”. |
| 1 | 5-The word "ın words" in line 62 should be corrected to "In the other words". | This has been corrected as suggested. |
| 1 | 6-The sentence starting with "In addition" in line 171 is not understood. | The sentence starting from “In addition…” has been rephrased for clarity as suggested. |
| 1 | 7-"The side effect" in line 207 this term is used in the medical field. Please write the sentence with another word. | This phrase has been replaced with “trade-off”. |
| 1 | 8- A word expressing possibility should be used instead of "should" in line 328. | This has been replaced with “is expected to”. |
| 1 | 10- Article spelling and grammar rules should be reviewed. | The comments related to the choice of words and sentence construction have been addressed accordingly. In addition, the article has been carefully proofread again as suggested. |
| 2 | The study was evaluated as a statistical method, and it was concluded that the proposed methods made significant contributions to the scientific method.  In the manuscript, especially in the discussion section, the importance of the statistical contribution of the proposed methods in terms of scientific research was not emphasized enough. In addition, it was observed that the literature support supporting the findings was not sufficient in the discussion and that recent studies were not cited as references. | The discussion has been revised according to the comment. Discussion has been arranged in a way to better highlight the findings, and to facilitate comparison with literature. Relevant recent papers have been cited accordingly.  In addition, for clarity and to better reflect the flow of text in the discussion, some parts of the results section have been rearranged. |
| 3 | To ensure that the manuscript meets the editorial and ethical standards of PLOS ONE, the following revisions are recommended:  -Please include a clear and specific ethics statement in the Methods section.  -Although the study is based on simulations and secondary data, PLOS ONE requires a formal ethics declaration. | A new section “Ethics” has been added as required, covering the ethical aspect of simulation and secondary data analyses. |
| 3 | - In accordance with the PLOS ONE data availability policy, authors must provide unrestricted access to the data and code underlying the findings. | This information is provided in PlosOne online submission form at Data availability section with a statement:  “The code and datasets used in this study are available at the following GitHub repository: <https://github.com/wnarifin/sipw_in_pvb>.”  We have also selected Yes to the question: *Do the authors confirm that all data underlying the findings described in their manuscript are fully available without restriction?*  In addition, the following texts have been added to experimental setup subsection:  “The code to generate and analyze the simulated data sets is available at https://github.com/wnarifin/sipw_in_pvb.”  “The clinical data sets and code to reproduce the results are available at https://github.com/wnarifin/sipw_in_pvb.” |
| 3 | -The simulation setup should be described in sufficient detail to allow full reproducibility. | The “experimental setup” section has been expanded into two subsections: simulation setup and analysis, clinical data analysis.  The names of the subsections in Results have also been revised to “simulated data analysis” and “clinical data analysis” to reflect these changes. |
| 3 | -While the results show improvements over existing methods, the Discussion section should explicitly acknowledge potential limitations. | The limitations are better highlighted in the Discussion section in the revised manuscript:  “While this study has demonstrated the strengths …” to “will be multiplied by 1000.” (the last paragraph in the discussion section) |

In addition, we have made some changes to the affiliation of authors.
